# An Overview of Poly(lactic-*co*-glycolic) Acid (PLGA)-Based Biomaterials for Bone Tissue Engineering

**DOI:** 10.3390/ijms15033640

**Published:** 2014-02-28

**Authors:** Piergiorgio Gentile, Valeria Chiono, Irene Carmagnola, Paul V. Hatton

**Affiliations:** 1 School of Clinical Dentistry, University of Sheffield, 19 Claremont Crescent, Sheffield S10 2TA, UK; E-Mail: paul.hatton@sheffield.ac.uk; 2 Politecnico di Torino, Department of Mechanical and Aerospace Engineering, Corso Duca degli Abruzzi 24, Turin 10129, Italy; E-Mails: valeria.chiono@polito.it (V.C.); irene.carmagnola@polito.it (I.C.)

**Keywords:** bone, composite, PLGA, scaffolds, tissue engineering

## Abstract

Poly(lactic-*co*-glycolic) acid (PLGA) has attracted considerable interest as a base material for biomedical applications due to its: (i) biocompatibility; (ii) tailored biodegradation rate (depending on the molecular weight and copolymer ratio); (iii) approval for clinical use in humans by the U.S. Food and Drug Administration (FDA); (iv) potential to modify surface properties to provide better interaction with biological materials; and (v) suitability for export to countries and cultures where implantation of animal-derived products is unpopular. This paper critically reviews the scientific challenge of manufacturing PLGA-based materials with suitable properties and shapes for specific biomedical applications, with special emphasis on bone tissue engineering. The analysis of the state of the art in the field reveals the presence of current innovative techniques for scaffolds and material manufacturing that are currently opening the way to prepare biomimetic PLGA substrates able to modulate cell interaction for improved substitution, restoration, or enhancement of bone tissue function.

## 1. Introduction

Bone tissue engineering is a research field with many clinical applications, such as bone replacement in the case of orthopaedic defects, bone neoplasia and tumours, pseudoarthrosis treatment, stabilization of spinal segments, as well as in maxillofacial, craniofacial, orthopaedic, reconstructive, trauma, and neck and head surgery [1–4]. For healing diseased or damaged bone tissue, the strategy of designing synthetic bone substitutes, called scaffolds, is a promising alternative to the use of allografts, autografts, and xenografts [5]. Scaffolds for bone repair should be based on biomaterials with adequate properties, such as biocompatibility, bioactivity, osteoconduction, osteoinduction, and biodegradation [5,6]. Among the materials, metals are suitable for load-bearing applications due to their favourable mechanical properties, whereas ceramics exhibit excellent biocompatibility as a result of their chemical composition that resembles the mineral phase of bone tissue. However, both metals and ceramics are generally poorly degradable [7]. In contrast, biodegradable polymers present appropriate characteristics, in terms of physico-chemical and biological properties, suitable to fabricate scaffolds for tissue engineering (TE) [8]. They can be classified as natural or synthetic. Natural polymers, such as proteins and polysaccharides, exhibit several benefits, such as degradability and negligible toxicity. However, a number of advantages are reported for synthetic polymers as compared with natural polymers, including the highly controlled and consistent degradation properties and excellent reproducible mechanical and physical properties such as tensile strength, elastic modulus and degradation rate [9]. Possible risks, such as toxicity, immunogenicity and favouring of infections, are lower for pure synthetic polymers with constituent monomeric units having a well-known and simple structure [10].

The most commonly used biodegradable synthetic polymers for three-dimensional (3D) scaffolds in tissue engineering are saturated poly(α-hydroxy esters), including poly(lactic acid) (PLA) and poly(glycolic acid) (PGA), as well as poly(lactic-*co*-glycolide) (PLGA) copolymers [11,12]. The chemical properties of these polymers allow hydrolytic degradation through de-esterification. Once degraded, the monomeric components of each polymer are removed by natural pathways. PGA is converted to metabolites or eliminated by other mechanisms, and PLA can be cleared through the tricarboxylic acid cycle. Due to these properties PLA and PGA have been used in biomedical products and devices, such as degradable sutures which have been approved by the US Food and Drug Administration as reported in Table 1 [13]. PGA is a hydrophilic and highly crystalline polymer with a relatively fast degradation rate. Although structurally very similar to PGA, PLA exhibits different chemical, physical, and mechanical properties because of the presence of a pendant methyl group on the alpha carbon. Generally, the co-polymer PLGA is preferred compared with its constituent homopolymers for the fabrication of bone substitute constructs, as PLGA offers superior control compared with degradation properties by varying the ratio between its monomers. PLGA, for instance, has a wide range of degradation rates, governed by the composition of chains, both hydrophobic/hydrophilic balance and crystallinity (Table 1) [14]. However, despite being biocompatible, clinical application of pure PLGA for bone regeneration is hampered by poor osteoconductivity and exhibits suboptimal mechanical properties for use as load-bearing applications. Therefore, PLGA is often used in combination with other materials, such as ceramics, bioactive glass, or it is opportunely modified in order to render PLGA more biomimetic and able to enhance bone regeneration [15].

In this review, the different applications of constructs based on PLGA for bone regeneration will be described, allowing a selection of the most promising solutions, as a base for future research in the bone tissue engineering field. Therefore, PLGA-based bone substitutes have been categorized according to its application forms: scaffolds, fibres, hydrogels or injectable microspheres. However, due to the wide variety of forms, the review will describe only composite constructs, based on PLGA and hydroxyapatite (HA), an inorganic filler largely used in bone tissue engineering due to its non-toxicity, bioactivity and osteoconductivity, and similarity to natural bone minerals [16]. Finally, the current trends in the development of functionalized PLGA constructs will be also reviewed, implying the use of conventional and innovative technique of functionalization.

## 2. Chemistry

### 2.1. Synthesis

**Figure 1.**
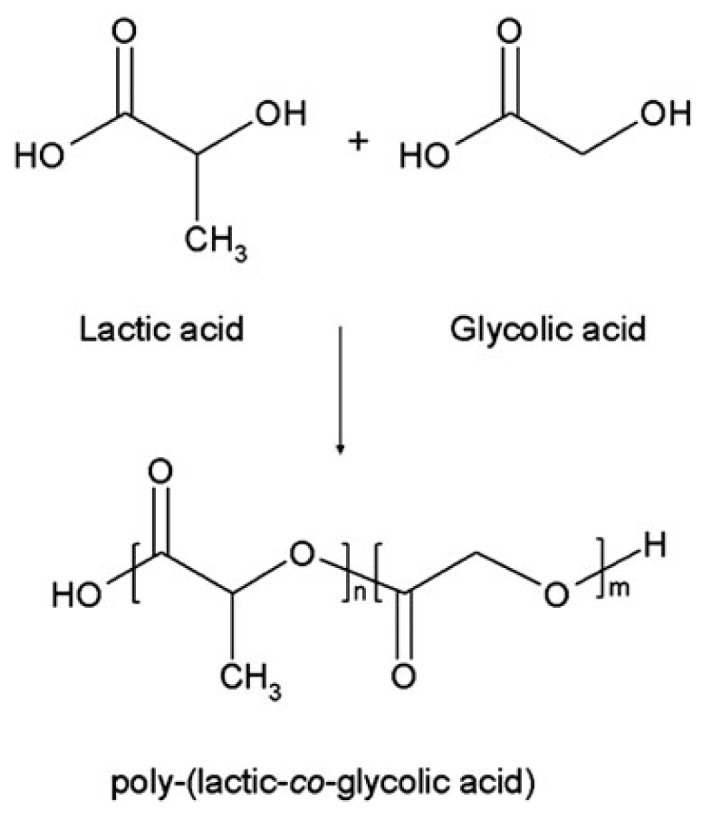
Chemical structure of poly(lactic-*co*-glycolic acid) and its monomers.

PLGA is a linear copolymer that can be prepared at different ratios between its constituent monomers, lactic (LA) and glycolic acid (GA) (Figure 1).

Depending on the ratio of lactide to glycolide used for the polymerization, different forms of PLGA can be obtained: these are usually identified in regard to the monomers’ ratio used (*i.e.*, PLGA 75:25 identifies a copolymer consisted of 75% lactic acid and 25% glycolic acid). Different synthesis mechanisms are used to obtain PLGA and the process parameters influence strongly the physico-chemical characteristics of the end product. Among them, the solution poly-condensation of LA and GA at temperatures above 120 °C under water-removal conditions allows the synthesis of PLGA with low molecular weight (*M*_W_ < 10 kDa) [17,18]. Ajioka *et al*. [19,20] reported a simple one-step method, a direct polycondensation from LA and GA, carried out in the azeotropical solvent diphenyl ether, characterized by a high boiling point. However, this solvent introduces higher levels of complexity of both process control and purification of the end product, and thus the resultant polymers are very expensive to produce. In 2001, Moon and Takahashi developed a new melt and melt/solid polycondensation process, without any azeotropical solvents, to overcome these drawbacks [21,22]. Moreover, high molecular weight PLGA could be obtained by ring opening polymerization, of lactide with glycolide using metal catalysts at high temperatures (130–220 °C), including tin (II) 2-ethylhexanoate, tin (II) alkoxides, or aluminum isopropoxide. Among these catalysts, the stannous octoate (SnOct2) is a highly efficient commercial catalyst and a food additive permitted in numerous countries [23,24]. An additional mechanism is represented by the enzymatic polymerization that appears as an alternative technique to obtaining aliphatic polyesters uncontaminated with possible toxic metallic residues, that is an essential prerogative for a synthesized material for biomedical applications. This mechanism of enzymatic ring-opening (*i.e.*, by lipase) occurs under mild reaction conditions (temperature, pH and pressure), but it needs a long reaction time, producing PLGA with a low molecular weight [25]. Usually, by the cited ring-opening polymerization technique, randomly distributed atactic or syndiotactic PLGA is obtained as reported in detail by Dechy-Cabaret [26], depending both on the monomer involved and on the course of the polymerization reaction. As known from literature, the PLGA sequence influences dramatically the degradation rate, because random PLGA degrades quicker than analog-sequenced PLGAs, prepared by ring-opening polymerization. Recently, a new method to obtain repeating-sequence PLGA copolymers with different tacticities has been proposed by Li *et al*. [27], using 1,3 diispropylcarbodiimide (DIC) and 4-(dimethylamino)pyridinium p-toluenesulfonate (DPTS) as catalysts. In this work, PLGAs with a high control over sequence and stereochemistry were produced, allowing for the tailoring and decrease of the hydrolysis rate. These results may be considered promising for the use of sequenced PLGA in biomedical applications, *i.e.*, in drug delivery applications, where the drug/biomolecule kinetic release is dramatically influenced by the polymeric degradation rate.

### 2.2. Physico-Chemical Properties

Unlike pure polylactic and polyglycolic acid show poor solubilities, PLGA can be dissolved by a wide range of common solvents, including chlorinated solvents, tetrahydrofuran, acetone or ethyl acetate [28] and it can be processed into any shape and size, and can encapsulate biomolecules of any size. Physical properties of PLGA have been shown to depend on different factors, including the initial molecular weight of the monomers, the LA:GA ratio, the exposure time to water and the storage temperature [29]. Table 1 show the physico-chemical properties and the field of application of different PLGA materials characterized by different LA:GA ratio.

As two enantiomeric isomers of lactide exist (e.g., d and l, according to the position of pendant methyl group on the alpha carbon of PLA), PLGA is available as d-, l-, and d,l-isomers. Although GA glycolic acid lacks the methyl side group (in contrast to LA), making it highly crystalline, PLGA copolymers are amorphous.

PLGA degrades by hydrolysis of its ester linkages, through bulk or heterogeneous erosion, in aqueous environments. In details, four steps can be described during its degradation: (i) hydration: water penetrates into the amorphous region and disrupts the van der Waals forces and hydrogen bonds, causing a decrease in the glass transition temperature (Tg); (ii) initial degradation: cleavage of covalent bonds, with a decrease in the molecular weight; (iii) constant degradation: carboxylic end groups autocatalyze the degradation process, and mass loss begins by massive cleavage of the backbone covalent bonds, resulting in loss of integrity; (iv) solubilization: the fragments are further cleaved to molecules that are soluble in the aqueous environment [30].

After the degradation, LA and GA are formed as by-products. The degradation rates can be influenced by different parameters: (i) the molecular weight: increasing the molecular weight of conventional PLGAs from 10–20 to 100 kDa, degradation rates were reported to range from several weeks to several months; (ii) the ratio of GA to LA: PLGA with a higher content of LA are less hydrophilic, absorb less water and subsequently degrade more slowly, as a consequence of the presence of methyl side groups in PLA making it more hydrophobic than PGA. An exception to this rule is the copolymer 50:50 which exhibits the faster degradation; (iii) stereochemistry: mixtures of d and l lactic acid monomers are most commonly used for PLGA fabrication, as the rate of water penetration is higher in amorphous d,l regions, leading to accelerated PLGA degradation; and (iv) end-group functionalization: polymers that are end-capped with esters (as opposed to the free carboxylic acid) demonstrate longer degradation half-lives [41,42]. Moreover, the shape of the device strongly affects PLGA degradation behaviour depending on the accessibility of water. In addition, acidic surrounding media accelerate PLGA degradation due to autocatalysis [43].

Finally, the Tg of the PLGA is reported to be above 37 °C and, hence, PLGA has a glassy behaviour in nature, showing fairly rigid chain structure. Furthermore, it has been described that Tg decreases with a decrease of LA content in the copolymer and with a decrease in the molecular weight [44].

## 3. Applications in Bone Tissue Engineering

For biomedical applications, PLGA has been used in a wide variety of forms, such as films, porous scaffolds, hydrogels, or microspheres that will be described in detail in the following paragraphs.

### 3.1. Porous PLGA-HA Scaffolds

Several techniques have been used to produce 3D porous scaffolds in the past decades, such as porogen leaching [45], gas foaming [46], phase separation [47], and solid freeform fabrication technologies [48]. Among these procedures, the porogen leaching has been taken by many groups, having the advantages of easy operation and providing an effective control of pore size and porosity, simply by varying the size and the amount of the porogens. Several papers in the literature concern the preparation of composite scaffolds, by particulate leaching, consisting of PLGA and bioceramic particles [49,50]. However, different disadvantages were reported: (i) the solvent removal by evaporation may be incomplete; (ii) it is more suited to producing thin scaffolds (up to 2 mm in thickness); and (iii) lack of interconnectivity and open-pore structure in scaffolds requiring a low porosity [51]. Kim *et al*. [52] described a novel method for fabricating a polymeric/nano-HA composite scaffold by gas forming and particulate leaching (GF/PL) without the use of organic solvents. The GF/PL method exposed HA nanoparticles at the scaffold surface significantly more than the conventional solvent casting/particulate leaching (SC/PL) method does. The GF/PL scaffolds showed highly porous structures, exhibited enhanced mechanical properties and significantly higher cell growth, alkaline phosphatase activity, and mineralization scaffolds *in vitro* compared to scaffolds fabricated by the SC/PL method. Moreover, different strategies were developed to allow the fabrication of 3D scaffold with a more complicated shape, combining the porogen leaching and the moulding technique together, avoiding high temperature of process, in order to reduce the degradation of PLGA during the processing. Cui *et al*. [53] reported a combination of the porogen leaching and melt-moulding, obtaining simultaneously an internal interconnected pore structure and an external complicated anatomical shape of the porous scaffolds. In their work, a PLGA, sodium chloride (NaCl) particle solutions, containing different amounts of HA, were first prepared by the conventional solvent casting, and then compressively moulded in a specially designed flexible-rigid combined mould, able to produce complicated scaffolds in shape. The composite solutions were then moulded under 10 MPa pressure at 150 °C (above the glass transition temperature but below the flow temperature of PLGA) for 5 min, and cooled to room temperature. Despite particulate leaching having been used in combination with other techniques, the lack of interconnectivity remained the major limitation [41].

**Figure 2.**
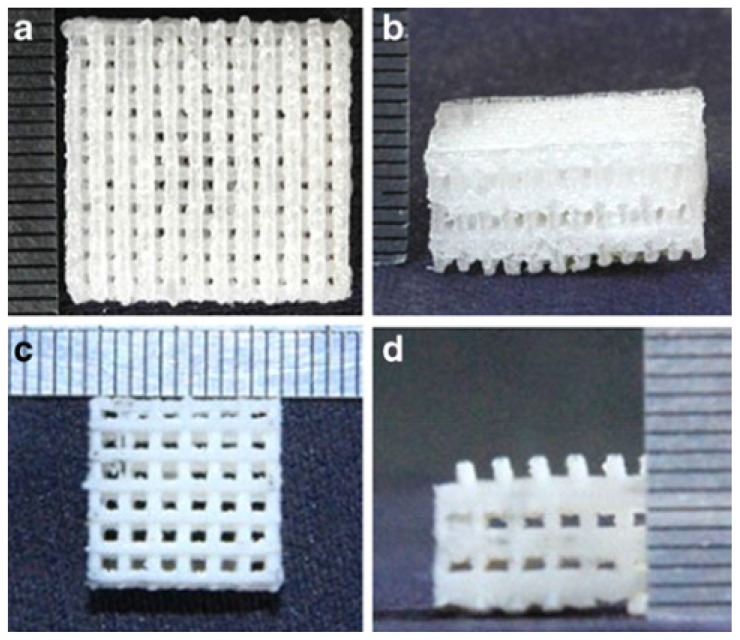
Porous scaffolds: (**a**,**b**) neat PLGA scaffold (top view and front view); (**c**,**d**) PLGA/nano-HA scaffold (top view and front view) [60].

Thermally induced phase separation (TIPS), based on changes in thermal energy to induce the de-mixing of a homogeneous polymer solution into a two or multi-phase system domain, has gained significant attention from a scientific and practical point of view. Currently, the TIPS technique is applied in the fabrication of microporous membranes or microcellular foams from medicine to the chemical industry, scaffolds for tissue engineering, and as a drug carrier for controlled release [54,55]. In 2011, Ebrahimian-Hosseinabadi prepared a biomimetic scaffold by TIPS at 60 °C, based on PLGA and a nano-biphasic component (nBCP), consisting of HA and β-tricalcium phosphate powders as reinforcement material. The authors described in detail the observed mechanical properties, showing that the composite with 20%–30% (*w*/*w*) of nBCP showed the highest and optimum value of yield strength and Young’s modulus among the scaffolds. Furthermore, it was observed that the agglomeration of reinforcing particles at higher percentages caused a reduction in mechanical properties [56]. Recent studies involved the solid freeform fabrication (SFF) technology to fabricate 3D scaffolds with a controlled internal/external micro-architecture, good accuracy, intricate internal pores, and complex geometries [57,58]. Among these techniques, evaluating the different process parameters and the physical properties of PLGA, the selective laser sintering (SLS) was found to be advantageous for TE scaffold fabrication, due to its ability to process a wide range of biocompatible and biodegradable materials [59]. In 2013, Shaui reported the preparation of PLGA/nano-HA composite porous scaffolds, with well-controlled pore architectures as well as high exposure of the bioactive ceramics to the scaffold surface, via selective laser sintering (Figure 2) [60]. The effect of nano-HA content on the microstructure and mechanical properties was investigated. The results showed that the compressive strength and modulus of the scaffolds were highly enhanced when the nano-HA content reached from 0 to 20% (*w*/*w*), while the mechanical properties decreased dramatically with an increased nano-HA amount [60].

### 3.2. Fibrous Scaffolds

The combination of enhanced mechanical properties, biocompatibility, and fibrous formability of the scaffolds is believed to have great potential for bone tissue regeneration. Different fibre-forming techniques were proposed in literature to fabricate micro- and nano-fibrous composite scaffolds [61]. The wet-spinning method was used by Morgan to obtain hollow fibres, as a scaffold, used in combination with human bone marrow stromal cells (HBMSCs) to initiate natural bone repair and regeneration [62]. The authors prepared a 20% (*w*/*w*) solution of PLGA (75:25) in 1-methyl-2-pyrrolidinone at 20 °C. Then, the polymer solution was passed through a spinneret (needle 0.3 mm outer diameter) and precipitated into a water coagulation bath, forming fibres with a diameter of the outer and inner walls around 770 and 500 μm, respectively. However, nanofibrous structures are more similar to that of the natural bone extracellular matrix (ECM), and may bring additional stimuli to the cultured cells. Electrospinning process represents a simple and versatile technology for producing ultrathin non-woven fibres with a diameter ranging from few nanometers to microns [63]. The electrospun nanofibres possess a lot of advantages, such as an extremely high surface-to-volume ratio, a small interfibrous pore size with tunable porosity, as well as wide possibilities for achieving desirable properties and functionalities [64]. Furthermore, in bone regeneration electrospun fibres are hypothesized as playing a role in sustaining mechanical properties, as well as allowing for biodegradability, and acting as an actual osteoconductive scaffold after addition or being coated by ceramic particles [65–67].

**Figure 3.**
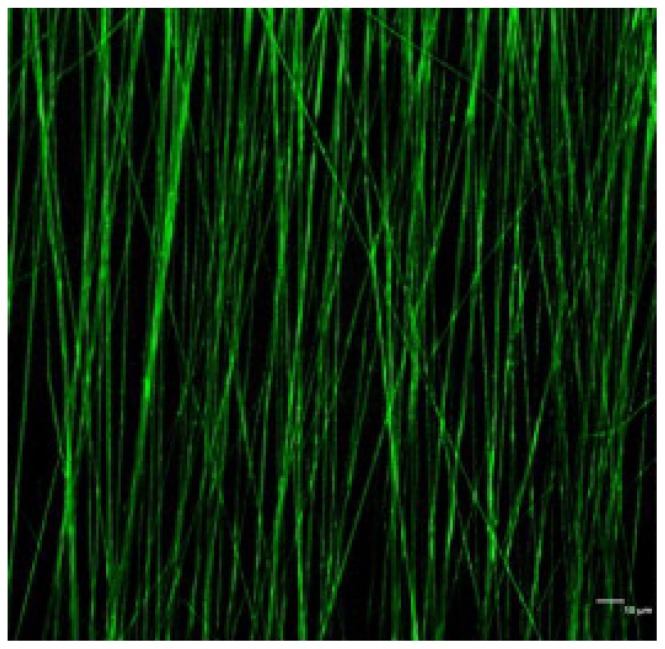
CLSM image showing dispersion of HA particles in PLGA fibres (Scale 10 μm) [72].

Several papers are present in literature, concerning the preparation of nanocomposite random fibrous scaffolds, in which HA has been added to the PLGA solution before spinning. Moreover, different process parameters were considered, such as the solvent (*i.e.*, dicloromethane, tetrahydrofuran, acetone), the polymeric concentration (from 15 to 25 *w*/*w*), the distance nozzle-collector (ranging from 10 to 25 cm), and, finally, the voltage (from 10 to 30 kV) [63,68,69]. The addition of an amphiphilic surfactant into the composite solution may reduce the agglomerate problems associated with the hydrophilic bioceramic powders within hydrophobic PLGA [70,71]. The physico-chemical properties of PLGA/HA electrospun scaffolds were carefully evaluated, considering different amounts of nano-HA. As reported in literature, Jose *et al*. [72] prepared aligned nanofibrous scaffolds based on PLGA and nano-hydroxyapatite (Figure 3), in which the inorganic filler concentration led to an increase in the glass transition temperature, causing a lower chain mobility, useful to prevent shrinkage.

Furthermore, nano-HA influenced strongly the mechanical properties, showing that the addition of nano-HA at 20% (*w*/*w*) influenced strongly the Young’s modulus of the electrospun composite meshes. However, the modulus decreased when the nano-HA concentration was increased from 20 wt %. The decrease in modulus may be due to poor interfacial properties between the particles and the polymer.

Recently, different research groups synthesized aligned nanofibrous scaffolds with promising results. Yun *et al*. [73] attempted to characterize the gene expression pattern during osteogenic differentiation of various stem cells on PLGA/nano-HA nanofibrous scaffolds (PLGA and nano-HA in a 5:1 blending ratio). They cultured primary adipose tissue-derived stem cells (hADSCs) and bone marrow cells (MSCs) from human in order to evaluate the biocompatibility of the scaffolds. The results showed that osteogenic differentiation and mineralization of stem cells cultured on the PLGA/HA nanofibre occurred. These results were confirmed by both an alkaline phosphatase activity (ALP) and a calcium assay. Moreover, they investigated whether osteogenic differentiation of stem cells affected the expression of osteogenic genes (osteocalcin, collagen type I). These gene mRNA and protein levels were found to increase in a time-dependent manner as confirmed by RT-PCR and Western blotting analysis.

### 3.3. Hydrogels

**Figure 4.**
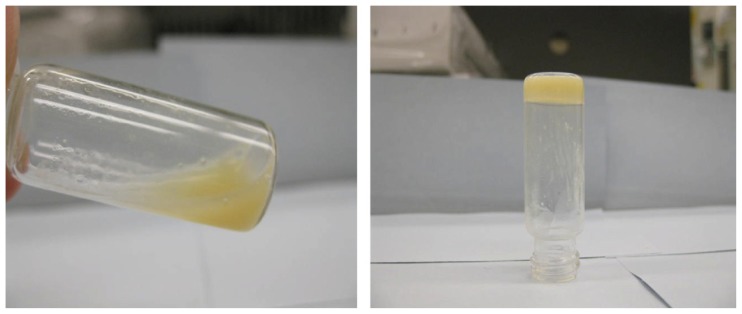
PLGA-g-PEG (30% *w*/*w*) hydrogel–HA composite containing 10% (*w*/*w*) HA was sol at 4 °C (**left**) and gel at 37 °C (**right**) [79].

Hydrogels are another class of scaffolds that are commonly used for tissue engineering applications. Hydrogels, such as fibrin, hyaluronic acid and Pluronic F127, have shown promise for effective growth factor delivery [74]. As reported by Dhillon *et al*. [75], blending PLGA with a plasticizer, such as poly(ethylene glycol) (PEG), allows the production of temperature-sensitive material with a reduced Tg of 37 °C. When the PLGA/PEG particles are mixed with a carrier solution at room temperature, a formulation is created that can be moulded or pasted at room temperature and, subsequently, hardens into a scaffold at 37 °C. The scaffold formation process starts with the particles becoming soft and cohesive when they reach their Tg, which causes them to adhere to each other. At this stage, the hydrophilic PEG component begins to leach out of the particles. This decrease in PEG content causes the Tg of the particles to increase, which results in them resolidifying. Strong adhesion bridges are formed between the fused particles, which create the PLGA/PEG scaffold structure. This scaffold system has recently been demonstrated to assist bone repair *in vivo* in a murine calvarial defect model [76]. However, there are drawbacks to use hydrogels for bone regeneration as they have low mechanical strength, which can hinder their individual use as bone replacements [77]. To overcome this issue, the combination of hydrogels and calcium phosphate particles is emerging as a well-established trend for bone substitutes. Besides acting as binders for the inorganic phase, hydrogels within these hybrid materials can modulate cell colonization physically and biologically [78]. Lin *et al*. [79] designed composites consisting of HA and a poly(ethylene glycol) (PEG) copolymer, namely PLGA-g-PEG (Figure 4). Interestingly, this was the only article reporting the viscoelastic profile as function of temperature. Concentrations of 30% *w*/*w* of hydrogel with different amounts of HA were analyzed. The results revealed that hydrogel composites preserved their sol-gel transition properties in the presence of HA. For all the formulations tested, the storage modulus displayed a maximum at 24 °C, dropping to values of <20 Pa at 37 °C. Moreover, the acidic pH environment of the hydrogel was neutralized by HA, both representing great advantages over the hydrogel alone. Scanning electron micrographs showed that HA particles were well dispersed and distributed within the hydrogel matrix. The composites showed a sustained release of a small molecule model dye for up to two weeks with slight increase of release with addition of HA.

### 3.4. Injectable Microspheres

Amorphous PLGA copolymers are suitable for biomedical applications, as provides a more homogeneous dispersion of the active species in the polymer matrix [80]. Due to the hydrolysis of PLGA, the effect of physico-chemical properties, such as the glass transition temperature (Tg), moisture content and molecular weight, on the rate of drug release from biodegradable polymeric matrices has been widely studied. The biodegradation properties of PLGA microparticles can be tailored from a few weeks to several months by varying the amount of poly(lactic acid) to poly(glycolic acid), influencing the release and degradation rates of incorporated drug molecules. The PLGA microspheres were fabricated by conventional oil/water emulsification method and different methods were proposed to obtain biomimetic injectable microspheres by addition of HA. Recently, negatively charged inorganic HA nanoparticles were assembled together with positively charged PLGA microspheres dispersed in deionized water to create a cohesive colloidal gel. This material was held together by electrostatic forces that may be disrupted by shear to facilitate extrusion, moulding, or injection. Scanning electron micrographs of the dried colloidal gels showed a well organized, three-dimensional porous structure. Rheology tests revealed that certain colloidal gels could recover after being sheared. Human umbilical cord mesenchymal stem cells were also highly viable when seeded on the colloidal gels [81]. Another approach was described by Kang *et al*. [82] that prepared HA-coated PLGA microspheres, previously prepared by water/oil/water, after immersion in simulated body fluid (SBF) for 5 days at 37 °C. The apatite-coated PLGA microspheres with osteoblasts were injected into a subcutaneous dorsum of the mice and tested for bone formation at 6 weeks. The new bone formation was significantly enhanced for the apatite-coated PLGA microsphere group compared to the plain PLGA microsphere group [82]. In another study, PLGA-hydroxyapatite microsphere composites were loaded with the bisphosphonate-based osteoporosis-preventing drug, alendronate (AL) [83]. These microspheres were prepared with the solid/oil/water or water/oil/water technique. The AL release from the PLGA/HA-AL system showed a sustained releasing tendency, except a minimal burst at the very beginning over a 30-day period. Their results indicated that the PLGA/HA-AL system was able to improve osteoblast proliferation, and, also, enable upregulation of ALP.

## 4. Current Trends in the Development of Functionalized PLGA Constructs

PLGA is widely used in bone tissue engineering due to its suitable physico-chemical properties and biodegradability rate; however, compared to natural polymerssuch as collagen and chitosan which have numerous ionic molecular groups, the synthetic polymers have relatively few of these groups, making it very difficult to induce mineralization [84,85]. Surface modification is an interesting approach to increase cell affinity or to generate a biomimetic interface between PLGA and the biological environmental. In this contest the main research goal is the formation of biomimetic apatite layer onto device surface [86]. As widely reported in literature, surface negative charge and topography influence the apatite formation [87,88]. Wan *et al*. [89] demonstrated that appropriate oxygen plasma treatment could not only incorporate functional groups on PLGA surface, increasing its negative charge, but also could change surface topography due to etching effects. Qu *et al*. [90] treated PLGA films and scaffolds with oxygen plasma and then incubated them in a modified SBF solution to obtain a bone-like apatite layer. The formation of bone-like apatite was enhanced by oxygen plasma treatment, allowing an increased number of negative charge and surface roughness. The formed apatite was similar in composition and structure to the natural bone mineral and enhanced the adhesion and proliferation of OCT-1 osteoblast-like cell when compared with neat PLGA.

Other strategies to functionalize the PLGA surface involve physical adsorption, a simple method to attach bioactive molecules, such as peptides and growth factor, but without controlling the amount and/or orientation of immobilized molecules. Using covalent techniques it is possible to obtain a better control of the amount immobilized, prolonged retention of the biomolecules, and ability to dictate the orientation/presentation of molecules [91]. Since Pierschbacher and Ruoslahti found that the argimine-glycine-aspatic acid (RGD) was the minimal cell-recognizable sequence in many extracellular matrix protein and blood proteins [92], the covalent linking of RGD to biomaterials is a widely used technique favouring their biological response. Yoon *et al*. [93] prepared a blend of PLGA and functionalized PLGA, in various ratios to prepare films by solvent casting or to fabricate porous scaffolds by a gas foaming/salt leaching method. In this work, carboxyl terminal end of PLGA was functionalized with a primary amine group by conjugating hexaethylene glycol-diamine. Then, under hydrating conditions, the activated GRGDY (Gly-Arg-Gly-Asp-Tyr) could be directly immobilized to the surface-exposed amine groups of the PLGA-NH_2_ blend films or scaffolds. The results showed that the extent of cell adhesion was substantially enhanced by increasing the blend ratio of PLGA-NH_2_ to PLGA. The level of an alkaline phosphatase activity (ALP), measured as a degree of cell differentiation, was also enhanced as a result of the introduction of cell adhesive peptides. Recently, Huang *et al*. [94] modified PLGA/nano-hydroxyapatite composite with GRGDSPC (Gly-Arg-Gly-Asp-Ser-Pro-Cys) through coupling mediated by a polyethyleneimine (PEI). The biocompatibility of PLGA/nano-HA-GRGDSPC was greatly increased compared with PLGA/nano-HA, and the scanning electron micrographs images showed that the cells grew better on PLGA/nano-HA-GRGDSPC both in 2D and 3D materials than that on PLGA/nano-HA. Moreover, the scaffolds PLGA/nHA-GRGDSPC showed better results in bone healing in rabbit mandibular defect.

**Figure 5.**
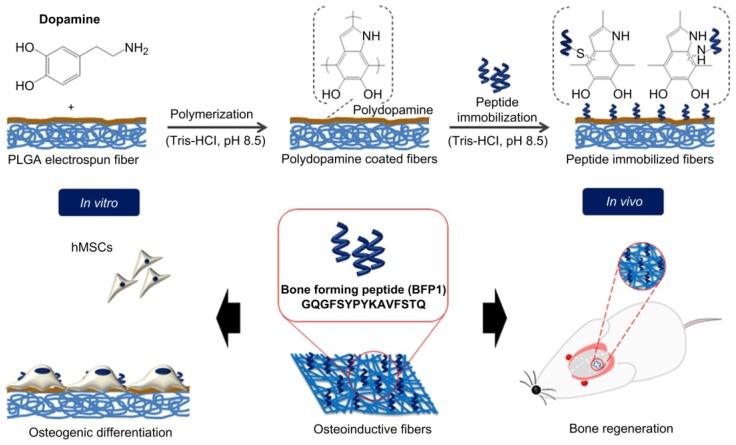
Schematic illustration of the preparation of electrospun fibres with immobilized BFP1 by polydopamine coating [98].

Unfortunately, the technologies for surface modification of synthetic polymers, such as covalent graft, ionized gas treatments, silane monolayers, generally require complex reaction conditions and specific equipment, which greatly limit the application at a large industrial scale [95]. An innovative approach to modify the synthetic polymer surface consists of employing mussel-inspired molecules,3,4-dihydroxy-l-phenylalanine (DOPA), that mimic the Mytilus edulis foot protein-5 (Mefp-5). DOPA and its derivates, such as 3,4-dihydroxyphenethylamine (dopamine) exhibit a powerful ability to adhere to almost any type of surface, including artificial polymers. The polymerization process of dopamine to polydopamine is likely to involve oxidation of the catechol (dopamine) to a quinone, followed by polymerization in a manner similar to that of melanin formation. Furthermore, the deposited film formed by polydopamine is easily adapted for a secondary modification due to it having many active functional groups [96,97]. Chen *et al*. [88] prepared a collagen/chitosan coating on polydopamine-modified PLGA in order to obtain a suitable material for guided bone regeneration (GBR). The composite membrane retained mechanical properties from PLGA and biological properties from natural polymer coatings. Recently, Lee and co-workers [98] fabricated barrier membranes with appropriate porosity and bioactivity for GBR, producing bioactive electrospun membranes based on PLGA by immobilizing bone-forming peptide 1 (BFP1) derived from the immature region of bone morphogenetic protein 7 (BMP7). The authors employed poly(dopamine) to graft BFP1 on nanofibre surfaces as shown in Figure 5.

*In vitro* tests performed using human mesenchymal stem cells (hMSCs) displayed a significant increase in ALP activity and calcium deposition when cells were cultured on BFP1 functionalized fibres. Moreover, *in vivo* tests carried out by implanting samples onto mouse calvarial defects, demonstrated an effective integration of the bioactive membranes with the host tissue.

## 5. Conclusions and Future Prospects

This article reviews the potential of PLGA to favour bone tissue engineering, due to its biological safety and tuneable degradation properties. As reported in this review, PLGA is categorized to its application forms: scaffolds, fibres, hydrogels or microspheres; composite constructs based on PLGA and hydroxyapatite are widely discussed. As reported, the addition of HA enhanced the osteoconductivity and the mechanical properties of PLGA scaffolds for their use as load-bearing applications, and the bone tissue regeneration. Finally, the review reports an alternative strategy to increase cell affinity or to generate a biomimetic interface between PLGA and the biological environment, involving the formation of the biomimetic apatite layer on the PLGA surface by surface modification. Furthermore, the combination of both strategy (HA addition and surface functionalization) in PLGA scaffold is expected to create an osteoconductive and osteoinductive gradient, allowing an increased success of bone tissue regeneration.

## Figures and Tables

**Table 1.** Properties and fabrication of biodegradable polymer materials.

Polymer	Modulus (GPa)	Elongation (%)	Solvent	Cristallinity (%)	Degradation Time (Weeks)	Applications	Reference
Polyglycolide/Polyglactine	7.0	15–20	Hexafluoroispropanol	45–55	6–12	Suture anchors, meniscus repair, medical devices, drug delivery, orbital floor	[31–33]
Poly(l-lactide)	2.7	-	Benzene, THF, dioxane	37	12–18	Fracture fixation, interference screws, suture anchors, meniscus repair	[33–35]
Poly(d,l-lactide)	-	3–10	Methanol, DMF	Amorphous	11–15	Orthopaedic implants, drug delivery	[32,33,36]
Poly(d,l-lactide-*co-*glycolide) 85/15	2.0	3–10	Ethyl acetate, chloroform, acetone, THF	Amorphous	5–6	Interference screws, suture anchors, ACL reconstruction	[33,34,37,38]
Poly(d,l-lactide-*co-*glycolide) 75/25	2.0	3–10	Ethyl acetate, chloroform, acetone, DMF, THF	Amorphous	4–5	Plates, mesh, screws, tack, drug delivery	[32,33,36]
Poly(d,l-lactide-*co-*glycolide) 50/50	2.0	3–10	Ethyl acetate, chloroform, acetone, DMF, THF	Amorphous	1–2	Orthopaedic implants, drug delivery	[36,38]
Poly (l-lactide-*co*-glycolide) 10/90	-						[39,40]
